# Identification of regulatory links between transcription and RNA processing with long-read sequencing

**DOI:** 10.1016/j.xpro.2023.102505

**Published:** 2023-09-20

**Authors:** Carlos Alfonso-Gonzalez, Laura Arrigoni, Hasan Can Ozbulut, Stefanie Falk, Ulrike Bönisch, Valérie Hilgers

**Affiliations:** 1 Max Planck Institute of Immunobiology and Epigenetics, 79108 Freiburg, Germany; 2 Faculty of Biology, Albert Ludwig University, 79104 Freiburg, Germany; 3 International Max Planck Research School for Molecular and Cellular Biology (IMPRS- MCB), 79108 Freiburg, Germany; 4 CIBSS Centre for Integrative Biological Signalling Studies, University of Freiburg, 79104 Freiburg, Germany

**Keywords:** Bioinformatics, Sequence Analysis, Genomics, Sequencing, RNAseq, Molecular Biology

## Abstract

We present a detailed protocol for sequencing full-length mRNA isoforms using the Oxford nanopore long-read sequencing technology. We describe steps for poly(A) RNA isolation, library preparation, and cDNA size selection. We then detail procedures for sequencing and processing and a computational framework to identify exon couplings and assign mRNA 5′ ends and 3′ ends to each other. Our approach enables the identification of links between transcription initiation and co-transcriptional RNA processing events.

For complete details on the use and execution of this protocol, please refer to Alfonso-Gonzalez et al.^1^

## Highlights

• Optimization of nanopore long-read sequencing samples for very long mRNAs
• Computational pipeline for the quantification of mRNA isoforms transcriptome wide
• Methods LASER and LATER identify couplings between transcription and RNA processing

## Abstract (editor highlights)

Publisher’s note: Undertaking any experimental protocol requires adherence to local institutional guidelines for laboratory safety and ethics.

## Graphical abstract

**Figure:**
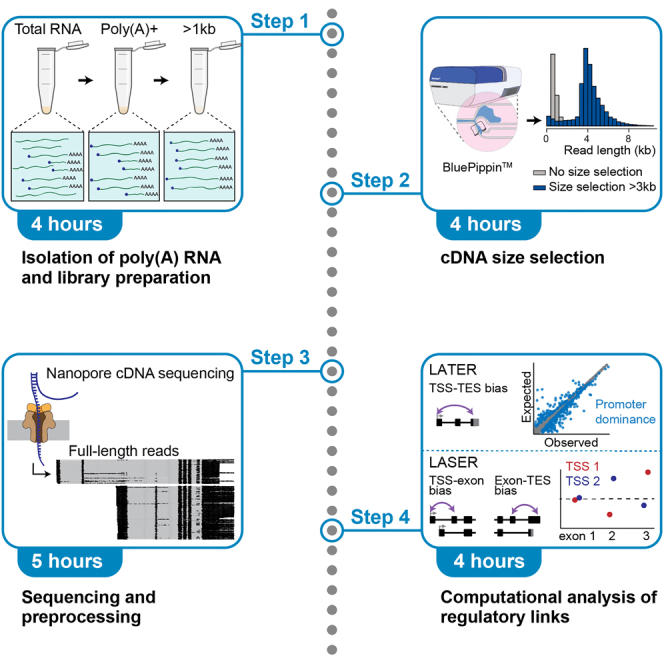

## Before you begin

The protocol below describes the specific steps for sequence analysis of long RNA fragments, including instructions for both sample preparation and computational analysis. We focus our analysis on the polyadenylated fraction of the transcriptome (Poly(A)+ RNA fragments).

Analysis of ultra-long RNA fragments absolutely depends on the preservation of long molecules throughout sampling and preparation steps. RNA is very susceptible to degradation by ribonucleases (RNases). Typical sources of RNase contamination are exposure to lab surfaces, aerosols from pipetting, ungloved hands or usage of contaminated reagents. Therefore, always wear gloves and exchange them frequently. Use RNase-free certified solutions and disposable plasticware, including filter tips. Use a separate, decontaminated area for RNA work and include RNase inhibitors into enzymatic reactions containing RNA fragments. Furthermore, keep RNA and thawed reagents on ice until needed and aliquot RNA samples to avoid multiple freeze-thaw cycles. Store RNA in RNase-free water or TE buffer at −85°C to −65°C for up to one year. Process only RNA of highest quality with no fragmentation detectable during initial quality assessment.

This protocol relies on a number of reagent kits and equipment for sample preparation, quality controls and sequencing. Make sure all reagent kits are available and devices including a sequencing platform from Oxford Nanopore Technologies (ONT) are installed and have been thoroughly tested. Depending on the sequencing platform in use, a high volume of data will be generated during sequencing runs. Platform-specific IT-requirements must be considered and trained bioinformaticians are needed for quality control, basecalling and data analysis.

## Key resources table

**Table:** 

REAGENT or RESOURCE	SOURCE	IDENTIFIER
**Critical commercial assays**		

Qubit RNA HS (high sensitivity) Assay Kit	Thermo Fisher Scientific	Q32852
Qubit RNA BR (broad range) Assay Kit	Thermo Fisher Scientific	Q10210
Qubit dsDNA HS (high sensitivity) Assay Kit	Thermo Fisher Scientific	Q33230
RNA kit (15NT), 500	Agilent Technologies	DNF-471-0500
HS RNA kit (15NT), 500	Agilent Technologies	DNF-472-0500
HS NGS fragment assay	Agilent Technologies	DNF-474-0500
HS Genomic DNA 50 kb Kit	Agilent Technologies	DNF-468-0500
Buffer EB	QIAGEN	19086
NEBNext Poly(A) mRNA Magnetic Isolation Module	New England Biolabs	NEB #E7490
Agencourt RNAClean XP	Beckman Coulter	A63987
Agencourt AMPure XP	Beckman Coulter	A63881
PCR-cDNA sequencing kit	Oxford Nanopore Technologies	SQK-PCS111
PCR-cDNA barcoding kit	Oxford Nanopore Technologies	SQL-PCB 111.24
0.75% agarose gel cassette with the external marker DF 3–10 kb S1	Sage Science	BLF7510
PromethION Flow Cell (R9.4.1)	Oxford Nanopore Technologies	FLO-PRO002
MinION and GridION Flow Cell (R9.4.1)	Oxford Nanopore Technologies	FLO-MIN106D

**Deposited data**		

*Drosophila* reference genome (dm6)	The FlyBase Consortium/Berkeley Drosophila Genome Project/Celera Genomics	https://www.ncbi.nlm.nih.gov/assembly/GCF_000001215.4/
Example datasets for analysis	This paper	https://github.com/ hilgers-lab /LATER/inst/extdatahttps://github.com/ hilgers-lab /LASER/inst/extdata
*Drosophila* Eukaryotic Promoter Database (EPD)	Meylan et al.^2^	N/A

**Software and algorithms**		

Long-reads-based Alternative Termination Estimation and Recognition (LATER) release v1.0.0	Alfonso-Gonzalez et al.^1^	https://github.com/hilgers-lab/LATER
Long-reads-based Alternative Splicing Estimation and Recognition (LASER) release v1.0.0	Alfonso-Gonzalez et al.^1^	https://github.com/hilgers-lab/LASER
R 4.1.1	R Development Core Team, 2021	https://www.R-project.org/
Minimap2 v2.17-r941	Li et al.^3^	https://github.com/lh3/minimap2
GenomicRanges_1.32.7	Lawrence et al.^4^	https://bioconductor.org/packages/release/bioc/html/GenomicRanges.html
GenomicFeatures_1.36.4	Lawrence et al.^4^	https://bioconductor.org/packages/release/bioc/html/GenomicFeatures.html
ggplot2_3.2.1	N/A	https://github.com/tidyverse/ggplot2
dplyr_1.0.8	N/A	https://github.com/tidyverse/dplyr
Rsamtools_2.10.0	N/A	https://bioconductor.org/packages/Rsamtools
SAMtools 1.12	N/A	git://github.com/samtools/htslib.git
DevTools	Wickham et al.^5^	https://cran.r-project.org/web/packages/devtools/index.html
Snakemake	Mölder et al.^6^	https://github.com/snakemake/snakemake
IGV	Robinson et al.^7^	https://software.broadinstitute.org/software/igv/
readr	Wickham et al.^8^	https://readr.tidyverse.org, https://github.com/tidyverse/readr
rtracklayer	Lawrence et al.^9^	https://bioconductor.org/packages/release/bioc/html/rtracklayer.html

**Other**		

Qubit Fluorometer	Thermo Fisher Scientific	Q33283
Fragment Analyzer system	Agilent Techologies	D0002110
BluePippin instrument	Sage Science	340BLU0001
Sequencing platform from Oxford Nanopore For instance, PromethION, GridION, or MinION	Oxford Nanopore Technologies	N/A Please refer to: https://nanoporetech.com/

## Materials and equipment

• 80% EtOH solution (10 mL stock): add 2 mL ddH_2_O to 8 mL EtOH.

***Note:*** Always prepare fresh and use within a day.

• 70% EtOH solution (10 mL stock): add 3 mL ddH_2_O to 7 mL EtOH.

***Note:*** Always prepare fresh and use within a day.

• Elution Buffer (10 mM Tris-HCl, pH 8.5, 100 mL): Add 1 mL 1 M Tris-HCl (pH 8.5) to 99.0 mL ddH_2_O.

***Note:*** Store at room temperature (20°C–25°C) for up to one year.

***Alternatives:*** Elution Buffer can be purchased at Buffer EB from Qiagen, Cat. No. 19086

• 0.1% Tween (10 mL): Add 0.1 mL 10% Tween 20 to 9.90 mL ddH_2_O.

***Note:*** Aliquot (1 mL) and store at 4°C for up to 6 month.

***Alternatives:*** Use 0.1% Tween solution provided in Kit BLF7510 from Sage Science

**Table:** TE Buffer

Reagent	Final concentration	Amount
Tris-HCl (pH 8.0), 1 M	10 mM	1 mL
EDTA (pH 8.0), 0.5 M	1 mM	0.2 mL
ddH_2_O	N/A	98.8 mL
**Total**	**N/A**	**100 mL**

Aliquot (1 mL) and store at 4°C for up to 12 month.

## Step-by-step method details

### RNA quality control

**Timing: 2 h**

Total RNA, isolated from the source of interest, must undergo rigorous quality checking including fluorometric quantification and fragment analysis (Fragment Analyzer, Bioanalyzer or TapeStationSystems from Agilent).

1. Fluorometric RNA quantification using the Qubit RNA HS (High Sensitivity, 0.2–200 ng/μL initial sample concentration) or BR (broad range, 0.5 to 1,200 ng/μL initial sample concentration) assay Kits. Troubleshooting 1.
  a. Dilute RNA samples with nuclease-free water to the assay-specific detection range.
    ***Note:*** The starting material is expected to have a relatively high RNA concentration, and using the Qubit broad range kit (0.5–2000 ng/μL) eliminates the need for sample dilution steps. In later stages of the protocol (following Poly(A) + RNA isolation and clean-up), the RNA concentration is anticipated to fall within the range of the high sensitivity RNA Qubit kit (0.2–200 ng/μL).
  b. Prepare the Qubit working solution by diluting the Qubit RNA Reagent 1:200 in Qubit RNA Buffer.
  c. To prepare the standards, add 10 μL of each Qubit standard to 190 μL Qubit working solution in Qubit assay tubes, mix by vortexing.
  d. To prepare RNA samples, add 1 μL of RNA sample to 199 μL Qubit working solution in Qubit assay tubes, mix by vortexing.
  e. Measure concentration in ng/μL using the Qubit fluorometer.
2. Fragment analysis using the Fragment Analyzer System from Agilent troubleshooting 2.
  a. Use the Agilent DNF-471 RNA Kit (15 nt, 5–500 ng/μL input sample concentration) or the Agilent DNF-472 HS (High Sensitivity) RNA (15 nt, 50–5,000 pg/μL input sample concentration) kit to perform automated capillary electrophoresis.
  b. Depending on the RNA concentration, dilute the samples using nuclease-free water to the detection range of the fragment analysis kit.
  c. Analyze RNA integrity by following the operating instructions of the Fragment Analyzer System and the ProSize data analysis software. Manuals are available online: https://www.agilent.com/cs/library/usermanuals/public/Fragment_Analyzer_system_manual_D0002110.pdf; https://www.agilent.com/cs/library/usermanuals/public/ProSize%20data%20analysis%20software%20user%20manual_D0002111_Rev_B.pdf.

**Figure 1:**
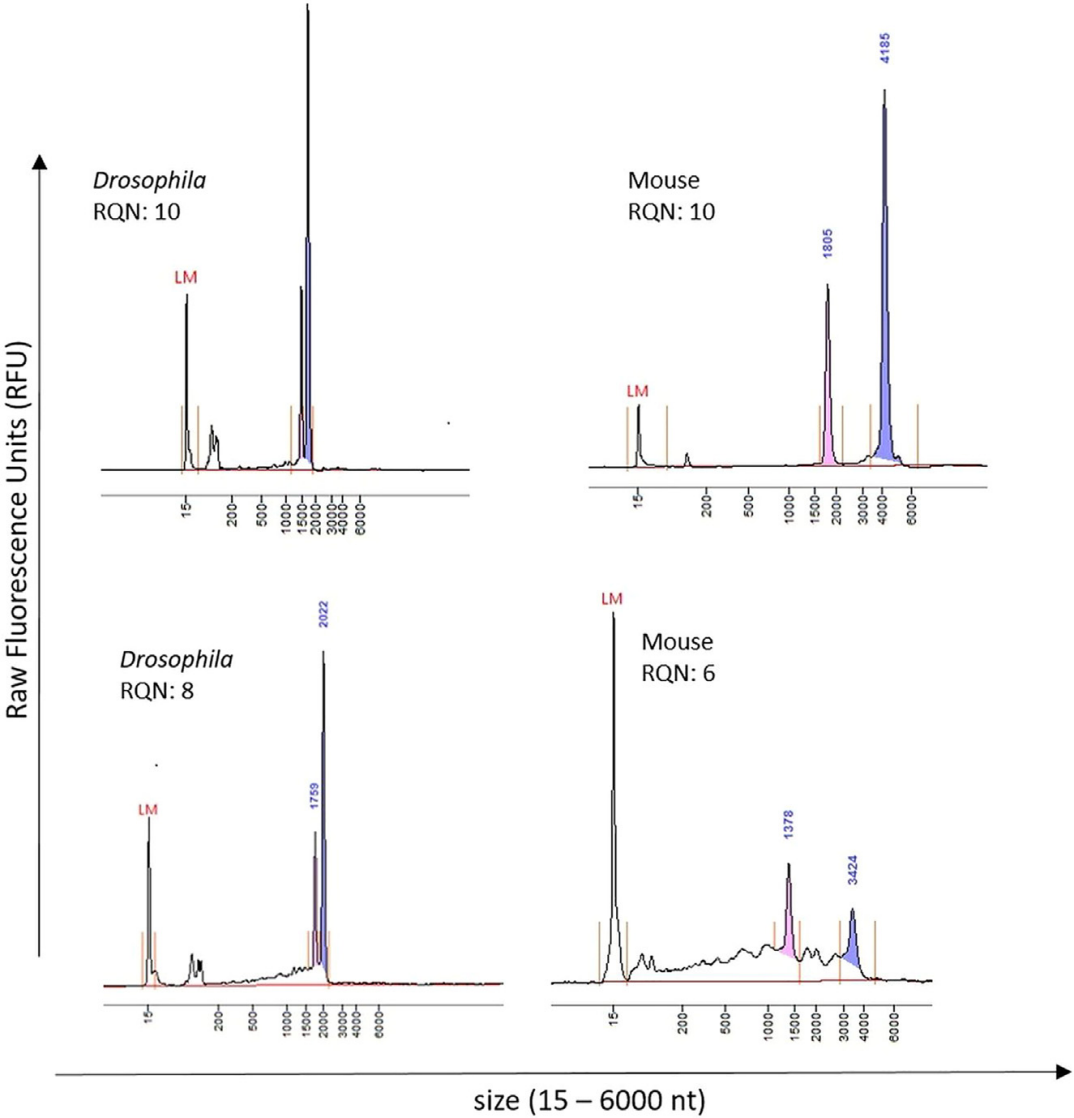
Total RNA fragment analysis and quality scoring Top panels: Total RNA isolated from *Drosophila* (left) or mouse (right), no fragmentation visible, RQN = 10. Lower panel: Total RNA isolated from *Drosophila* (left) or mouse (right), smeary baseline indicates RNA fragmentation, RQN = 8 or 6, not useful for detection of ultra-long transcript isoforms (> 10 kb). LM: Lower marker needed for size calibration, Blue and pink: ribosomal RNA fractions (28S and 18S) recognized by the software to calculate RNA Quality Numbers (RQN), 1 (poorest), 10 (highest) quality.

Use RNA Quality Numbers (RQN) for data interpretation. Figure 1 shows RNA fragment analyses, demonstrating optimal RNA quality parameters across various species.

**CRITICAL:** Use only total RNA preparations of very high quality with no RNA fragmentation visible (flat baseline of electropherogram). RQN/RIN should be > 9. (RIN: RNA Integrity Number if Bioanalyzer or TapeStation systems are used for RNA qualification)

### Selection of poly(A)+ RNA

**Timing: 2 h**

Poly(A)+ RNA transcripts are selected from DNA-free total RNA preparations using Oligo d(T)-coupled paramagnetic beads. Use the NEBNext Poly(A) mRNA Magnetic Isolation Module from NEB (E7490). All reagents within the module should be stored at 4°C. Reagent expiry dates can be found on each tube. The module provides a step-by-step protocol for isolating Poly(A)+ RNA from total RNA preparations, which should be followed in detail as the selection of Poly(A)+ RNA increases library preparation efficiency and enables sequencing of full-length RNAs at a later stage.

3. Prepare several DNA-free total RNA aliquots in PCR tube strips containing 4 μg of high-quality total RNA each. According to the Poly(A)+ selection module, starting material for Poly(A)+ isolation can vary from 1 to 5 μg of total RNA. The RNA should be diluted in a final volume of 50 μL nuclease-free water.

***Note:*** To generate sufficient material for a single experiment, we found eight 4-μg total RNA aliquots sufficient for an experiment run using a MinION/GridION or even a PromethION sequencing platform.

4. Perform Poly(A)+ RNA isolation by following instructions of the NEBNext Poly(A)+ mRNA Magnetic Isolation module. https://international.neb.com/-/media/nebus/files/manuals/manuale7490.pdf?rev=8fc35eb8aee94d0b90f0b671aa5a46ac&hash=C8CCC69263E3FEA33B00126133F6A118.
5. Measure Poly(A)+ RNA concentration and analyze RNA fragment size. Perform quality control by following the instructions detailed under step 1 “RNA quality control”.

**Figure 2:**
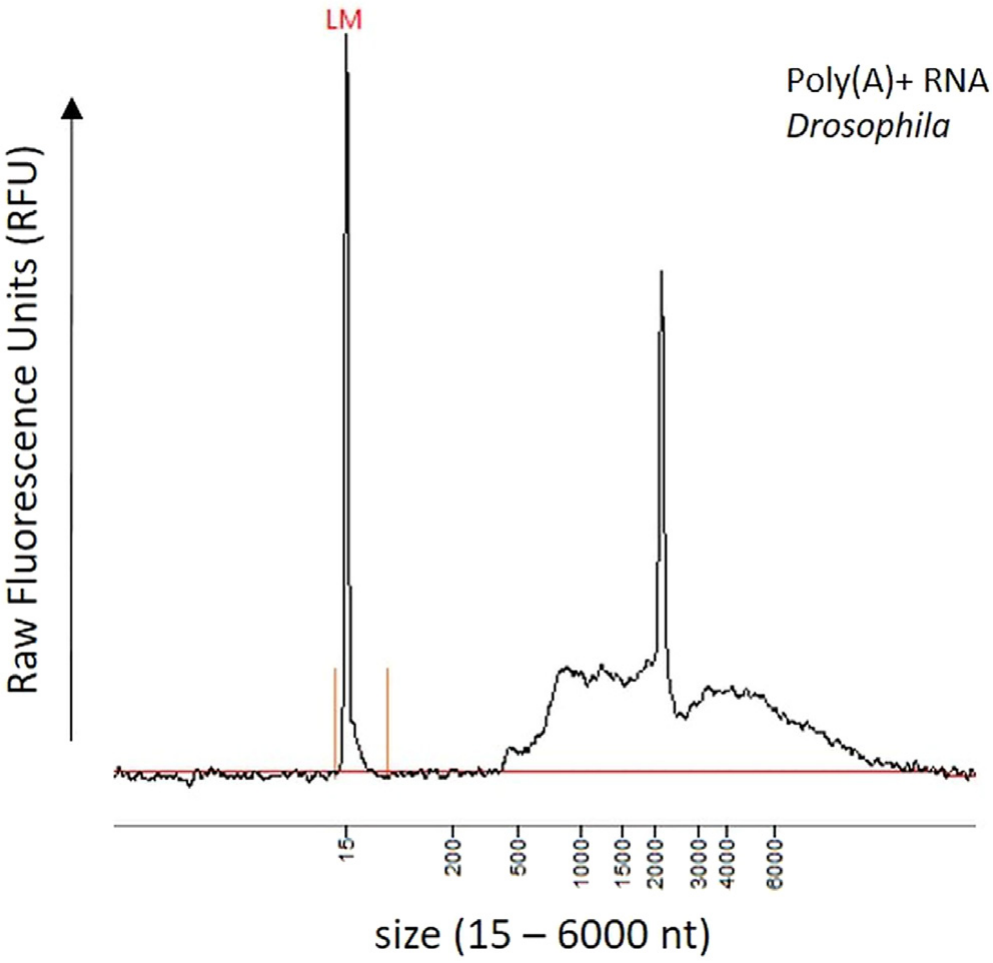
Poly(A)+ RNA fragment analysis The NEBNext Poly(A) mRNA Magnetic Isolation Module from NEB was used to isolate Poly(A)+ RNA from total RNA preparations. Samples were analyzed on a Fragment Analyzer system using the Agilent DNF-471 RNA Kit (15 nt). LM: Lower marker needed for size calibration.

***Note:*** Software assisted assessment of RNA fragmentation level (RQN/RIN) is not possible at this step, since ribosomal RNA signals are removed post Poly(A)+ enrichment. Hence, only a visual inspection of the traces is possible. After Poly(A)+ enrichment, typically RNA fragments are visible beyond 500 nt. Enrichment of fragments smaller than 500 nucleotides can be interpreted as sample fragmentation. Poly(A)+ RNA extracted from drosophila typically shows a prominent signal around 2000 nt (Figure 2).

**CRITICAL:** Keep samples and all the reagents used during the Poly(A) isolation, except the NEBNext Oligo d(T)_25_ beads, on ice when not in use.

### Exclusion of short Poly(A)+ RNA fragments (<1 Kb)

**Timing: 2 h**

6. Exclude short RNA fragments using Agencourt RNAClean XP beads **(upon arrival, aliquot RNACleanXP beads into 1 mL aliquots and store at 4°C for up to 18 months)**.
  a. Bring Agencourt RNAClean XP beads to room temperature (30 min at 20°C–25°C).
  b. Prior to usage, vortex Agencourt RNAClean XP beads vigorously.
  c. If multiple Poly(A)+ RNA fractions were prepared, pool fractions prior to bead clean-up.
  d. Use a bead-to-sample ratio of 0.4. For example, starting with a Poly(A)+ RNA sample volume of 50 μL, add 20 μL beads.
  e. Mix Poly(A)+ RNA sample and Agencourt RNAClean XP beads and incubate for 5 min at room temperature to bind RNA to magnetic beads.
  f. Place on a magnetic stand, wait until supernatant is clear Remove and discard supernatant.
  g. While keeping tubes on a magnetic rack, add 200 μL of 70% EtOH (freshly prepared) without disturbing the beads.
  h. Remove the ethanol and repeat the wash step while keeping the tubes on the magnet.
  i. Remove any residual Ethanol and dry beads for maximum 30 s while keeping the cap of the tubes open.
  j. Remove tubes from the magnet and add 20 μL elution buffer (10 mM Tris-Cl, pH 8.5) to elute Poly(A)+ RNA from the beads.
  k. Mix well by pipetting and incubate at room temperature for 2 min.
  l. Place tubes on a magnetic stand and wait until supernatant is clear.
  m. Transfer supernatant, containing the sample, into clean tubes. Keep tubes on ice until cDNA generation.
7. Measure RNA concentration using Qubit HS RNA assay following manufacturer’s instructions.

**Figure 3:**
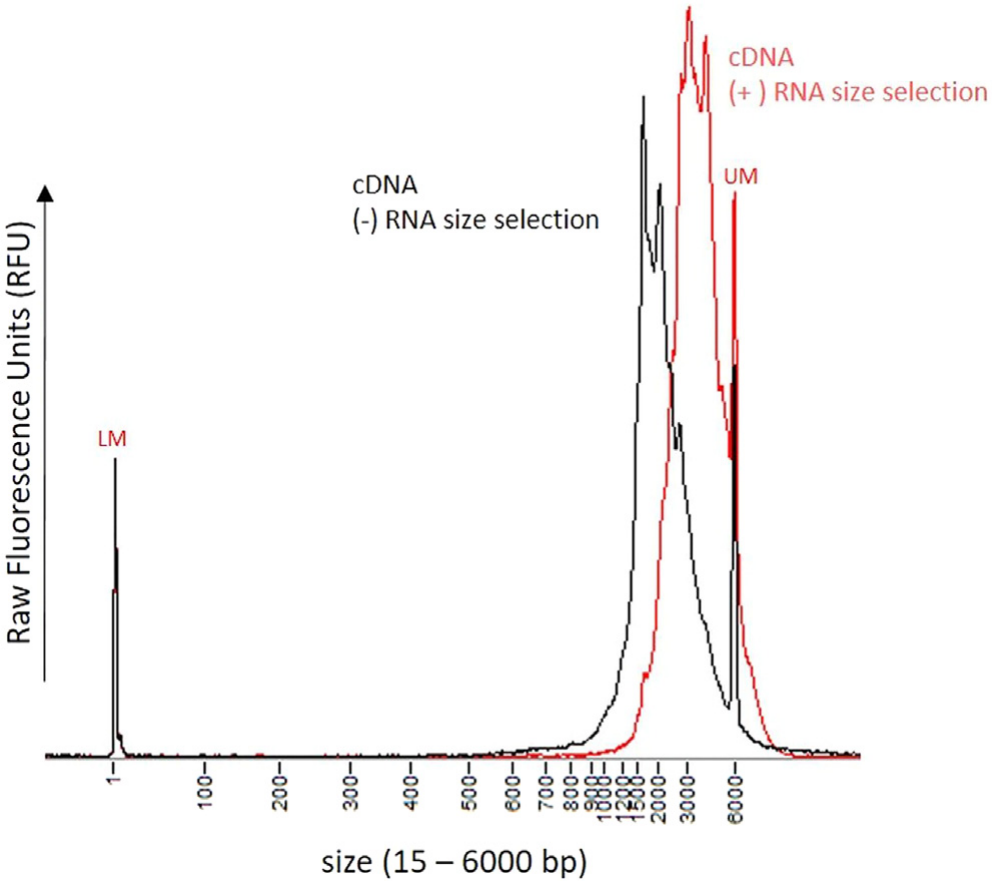
Fragment analysis of cDNA (after library preparation) Black line: Poly(A)+ RNA was not size selected, Red line: Poly(A)+ RNA fragments < 1 kb were depleted prior to cDNA preparation. Samples were analyzed on a Fragment Analyzer system using the HS NGS Fragment assay reagents. LM/UM: Lower / Upper Marker needed for size calibration.

***Optional:*** assess size distribution by capillary electrophoresis to visualize small molecule-depleted Poly(A)+ RNA. This control is informative to check whether any RNA degradation occurred in the process. At this point, the loss of small RNA fragments is not yet visible and can be only visualized after cDNA synthesis (Figure 3).

**CRITICAL:** To work with Agencourt RNAClean XP and AMPure XP cleanup magnetic beads, use low binding or low retention filter tips to avoid sample loss. Most plasticware providers offer low retention filter tips that can be used throughout the course of the experiment to avoid sample loss.

### Generation of cDNA nanopore sequencing library

**Timing: 7 h**

Use the latest cDNA library preparation protocol from Oxford Nanopore https://community.nanoporetech.com/docs/prepare/library_prep_protocols.

***Note:*** To assess online support, a login is required. The link refers to all Oxford Nanopore library preparation protocols. Prior to protocol download, the sequencing instrument must be specified, to include device-specific instructions: https://community.nanoporetech.com/docs/prepare/library_prep_protocols

Depending on the scale of the experiment and the sequencing platform, choose the workflow with or without sample barcoding and follow the step-by-step protocol (PCR-cDNA Barcoding Kit (SQL-PCB 111.24) or PCR-cDNA Kit (SQK-PCS111). Store reagents of the library preparation kit at −20°C. Expiry dates are printed on each tube. To maximize read length, include the optimized steps detailed below:

8. Use 4 ng size-selected Poly(A)+ RNA in 9 μL nuclease-free water (input recommendation of the library preparation kit). Perform multiple preparations in parallel to collect enough material for gel-based size selection.
9. Split each 20 μL reaction into 4 PCR reactions using 5 μL reverse-transcribed RNA as input.
10. Amplify each reaction using the cycling conditions detailed in SQK-PCB111.24 but change the extension time to 16 min (60 s/kb).

***Note:*** The number of PCR cycles can vary between 10 and 18. We recommend using 16 cycles as a starting point, since enough material for gel-based size selection must be prepared. The number of PCR cycles can negatively impact read length, which results in a decrease of N50 values. In our experiments, we have not reduced PCR cycles below 16, so amplification down to 14 or even 10 cycles should be tested case by case.

***Note:*** Due to long extension times (16 min/cycle), the PCR will last up to 5 h. We typically set up the PCR overnight and leave the reaction at 4°C prior to AMPure XP clean up.

**Table:** PCR cycling conditions

Steps	Temperature	Time	Cycles
Initial Denaturation	95°C	30 s	1
Denaturation	95°C	15 s	16 cycles
Annealing	62°C	15 s	
Extension	65°C	16 min	
Final extension	65°C	6 min	1
Hold	4°C	forever

11. Pool all the processed fractions into a 1.5 mL tube. Clean and size select the sample using AMPure XP beads **(upon arrival, aliquot AMPure XP beads into 1 mL aliquots and store at 4°C for up to 18 months)** , with bead-to-sample ratio of 0.4.
  ***Note:*** after pooling, the volume of the resulting sample to clean up can be relatively high. For instance, if library preparation has been started with four poly(A)+ RNA size selected replicates and each replicate split into four for the PCR step, a volume of 800 μL should be expected (16 samples, 50 μL each). In this example 320 μL of AMPure XP beads have to be used to clean this pool.
  a. Bring AMPure XP beads to room temperature (30 min at 20°C–25°C).
  b. Vortex AMPure XP beads vigorously.
  c. Add 20 μL of beads per every 50 μL of sample volume.
  d. Mix sample and AMPure XP beads by vortexing and incubate for 5 min at room temperature to bind cDNA to magnetic beads.
  e. Place on a magnetic stand, wait until supernatant is clear.
  f. Remove and discard supernatant.
  g. While keeping tubes on a magnetic rack, add 200 μL of 80% EtOH (freshly prepared) without disturbing the beads.
  h. Remove the ethanol and repeat the wash step while keeping the tubes on the magnet.
  i. Remove any residual Ethanol and dry beads for max. 30 s while keeping the cap of the tubes open. Remove tubes from the magnet and elute amplified cDNA in not more than 30 μL TE buffer.
  j. Mix well by pipetting and incubate at room temperature for 2 min.
  k. Place tubes on a magnetic stand and wait until supernatant is clear.
  l. Transfer supernatant containing the size-selected cDNA, into clean tubes. Keep tubes on ice until cDNA generation.
12. Elute amplified cDNA in not more than 30 μL TE buffer.
13. Asses yield (fluorometer) and size distribution (capillary electrophoresis) of amplified, full-length cDNA (Figure 3).
14. DNA quality control: for quantification, use the Qubit dsDNA HS Assay. For fragment analysis, use the HS NGS Fragment assay (DNF-474-0500, 100–6,000 bp) or the HS Genomic DNA 50 kb Kit (DNF-468-0500, 75–60,000 bp).

**Pause point:** After library preparation, samples can be stored overnight at 4°C or for up to 4 weeks at –20°C.

### Enrichment of cDNA fragments larger than 3 kb

**Timing: 4 h**

The BluePippin instrument is an automated DNA size selection system. Using pre-cast gel cassettes with different agarose concentrations, selection of DNA fragments between 100 bp – 50 kb is supported. The section below describes settings for selection of cDNA fragments larger than 3 kb.

**Figure 4:**
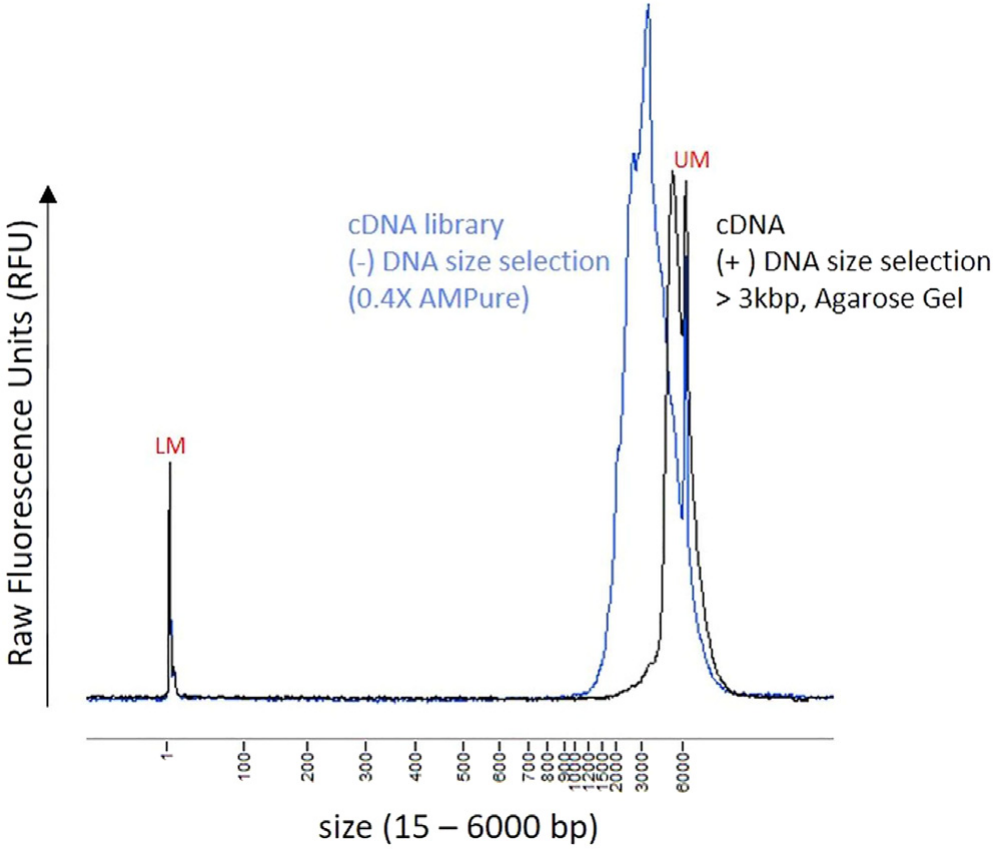
Fragment analysis of cDNA (after library preparation) Blue line: Amplified cDNA cleaned using 0.4× AMPure beads, Black line: Amplified cDNA size selected using agarose gel on a BluePippin Instrument. Samples were analyzed on a Fragment Analyzer system using the HS NGS Fragment assay reagents (1–6000 bp). RFU: Raw fluorescence value, LM/UM: Lower/Upper Marker needed for size calibration.

15. Perform DNA size selection using a 0.75% Agarose Gel Cassette with the external marker DF 3–10 kb S1.
  ***Note:*** Pre-cast gels are stored at room temperature, provided reagents (marker and electrophoresis buffer) are stored at 4°C. Expiry dates are given on each gel cassette and reagent tube.
  a. Bring DNA sample up to 30 μL in TE buffer (max. 5 μg DNA).
  b. Add 10 μL loading solution to each sample and mix samples thoroughly, centrifuge to collect sample.
  c. Program a protocol as described in the reference guide of the BluePippin software.
  d. Enter a base pair threshold (BP Start) for DNA size selection: 3 kb.
  e. Enter a base pair threshold (BP End) for DNA size selection: 15 kb.
    ***Note:*** BP end values do not represent the true upper limit of collection. All fragments > 3 Kb will be collected. The value (BP end) is required for the software to function properly.
  f. Determine the lane to which the external marker S1 (3–10 Kb) will be added.
  g. After run completion, wait for 45 min with closed lid to collect 40 μL of the sample out of the cassette and transfer into a 1.5 mL tube (fraction 1).
  h. Perform a second elution with a 0.1% Tween solution (also provided in the kit). Before pipetting out the second eluate, wait for 10 min and transfer 40 μL of sample into a fresh 1.5 mL tube (fraction 2).
    **CRITICAL:** Samples are either in a Tris-TAPS buffer (fraction 1) or in 0.1% Tween solution (fraction 2). Both Tris-TAPS buffer and 0.1% Tween are not suitable for nanopore sequencing, therefore the buffer must be exchanged through an AMPure XP cleanup (see step below).
16. Perform an additional AMPure XP cleanup.
  a. Combine cDNA fractions of the same sample and mix by vortexing, with equal volume of AMPure XP beads.
  b. Incubate for 5 min at room temperature to bind cDNA to magnetic beads.
  c. Place on a magnetic stand, wait until supernatant is clear.
  d. Remove and discard supernatant. Elute the cDNA in 12 μL Oxford Nanopore elution buffer (EB).
17. For each sample, analyze 1 μL of the amplified cDNA for size, quantity and quality using a Qubit fluorometer and a Fragment Analyzer (Figure 4).

### Loading of the nanopore flow cells

Follow instructions of the PCR-cDNA library preparation kit for adapter ligation, flow cell priming and loading, as well as for data acquisition parameters and basecalling.

***Note:*** Oxford Nanopore offers sequencing platforms scalable in size. The number of nanopores per flow cell vary and define the maximal output measured in number of reads or number of sequenced bases. SpotON flow cells (compatible with MinION and GridION devices) hold 512 active nanopores and, on average, 10 GB data can be produced per run. PromethION flow cells (compatible with PromethION devices) hold 5000 nanopores and can produce an average of 100 GB of data per run. Based on our experience, one million reads with an average size of 3 Kb (3 GB of data) is sufficient to detect a significant number of genes and couplings (Figure 8). Therefore, either MinION, GridION, or PromethION instruments can be used. Sequencing on a PromethION platform is typically the most cost-efficient since the price per GB sequenced data is comparably lower and sample multiplexing is also more feasible.

18. Load samples. Instructions differ for GridION/MinION or PromethION sequencing platforms:
  a. For PromethION flow cells, load 15–25 fmols of (barcoded) amplified cDNA in 23 μL Elution Buffer from Nanopore.
  b. For SpotON flow cells (GridION and MinION) load 15–25 fmols of (barcoded) amplified cDNA in 11 μL Elution Buffer from Nanopore.
  c. For Mass to Moles conversion of dsDNA, the following webpage is useful: https://nebiocalculator.neb.com/-!/dsdnaamt.

***Note:*** Oxford Nanopore released R10.4 flow cells on 01/2023, improving read accuracy. Up to now, not all kits are compatible with the newly released flow cells. SQK-PCB111.24/SQK-PCS111 library preparation kits are currently only compatible with previously used R9.4.1 flow cells.

**Figure 5:**
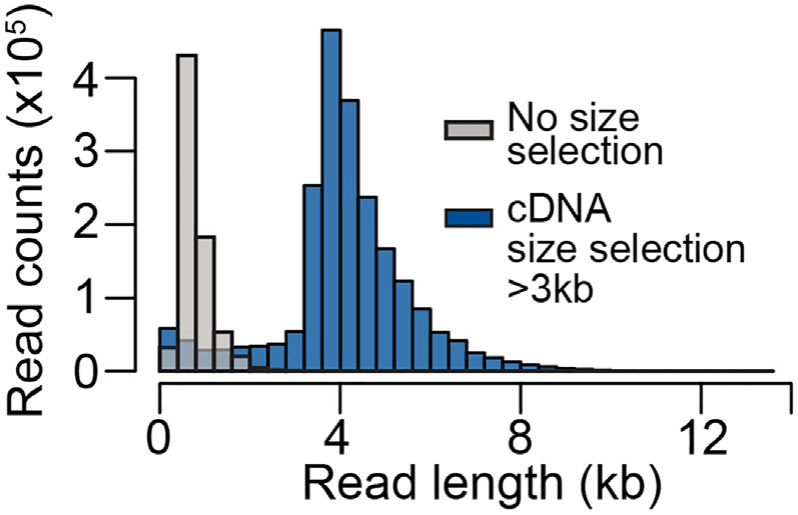
Histogram representing the read lengths from long-read sequencing data before and after the size selection procedure Note that the increase in longer reads corresponds to the threshold used for size selection.

After sequencing, the mapped reads should show a clearly shifted size distribution compared to the non-size selected samples (for an example, see Figure 5).

## Expected outcomes

**Figure 6:**
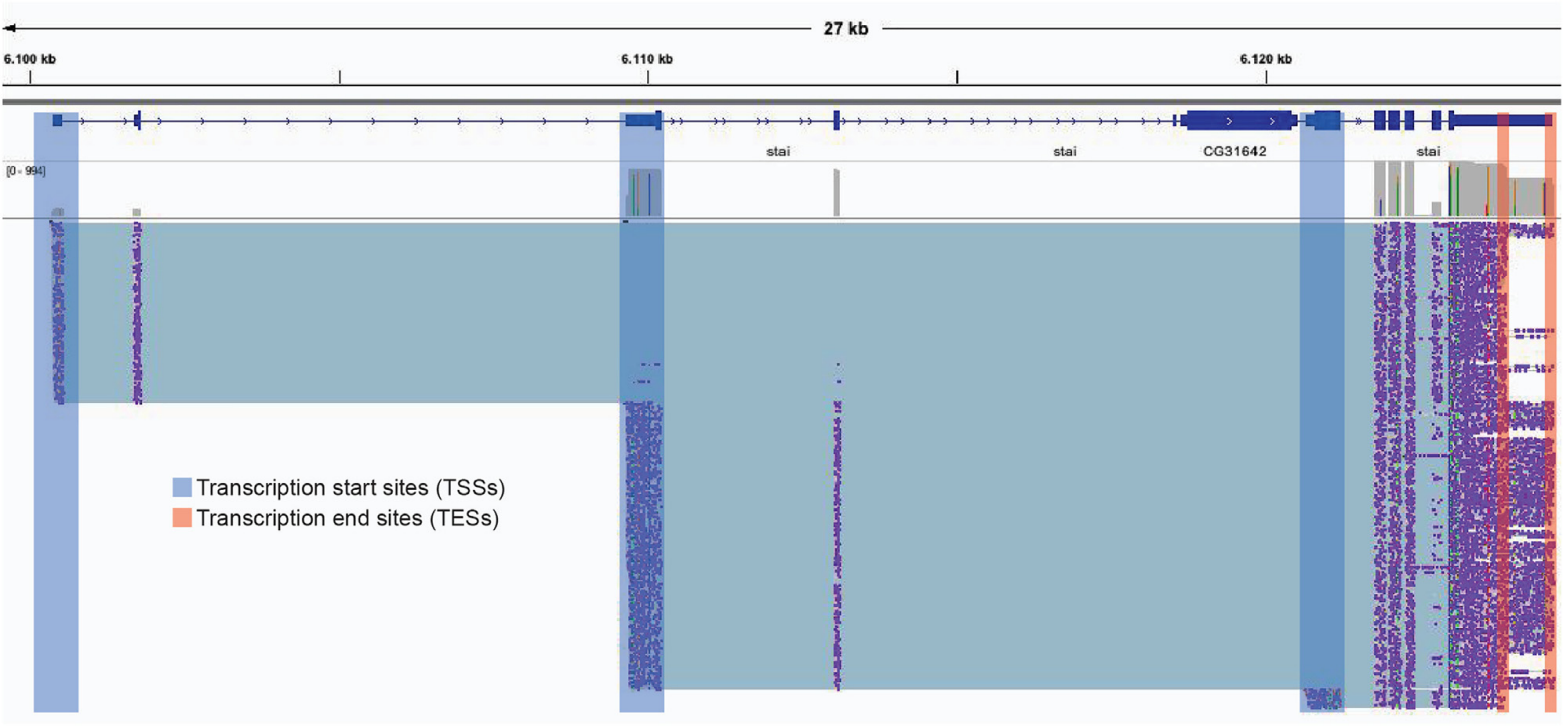
IGV screenshot from long-read sequencing data Each horizontal line represents a single read. In boxes, TSSs (blue) and TESs (red) used in LATER for the filtering of reads spanning both features.

**Figure 7:**
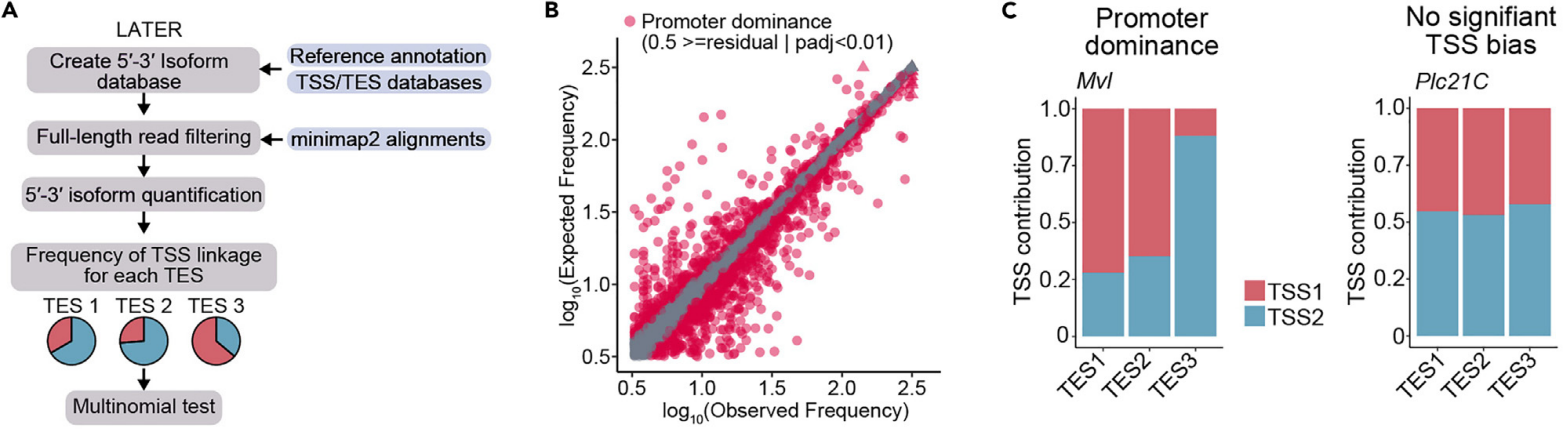
LATER quantifies couplings between TSSs and TESs (A) LATER Workflow illustrating each step of the pipeline. Auxiliary files required for each step are highlighted in blue. (B) Scatterplot showing the comparison between expected and observed frequencies of quantified 5′–3′ isoforms, a calculation obtained using LATER. The 5′–3′ isoforms that pass the residual cut-off and are statistically significant (p < 0.01, chi-squared test with Monte Carlo simulation and Benjamin-Hochberg correction) are highlighted in red. (C) Contribution of the TSS to the expression of each 3′ end (at alternative TESs) of the gene. Genes exhibiting promoter dominance display an imbalance in the production of 3′ ends, favoring certain ends over others. TSSs in genes without bias produce all 3′ ends at equal proportions.

We anticipate that this method will facilitate the characterization of RNA processing events occurring within individual RNA molecules using long-read sequencing. By combining our optimized experimental long-read sequencing protocol with computational pipelines, we can quantify and identify these couplings. We employed multinomial testing within the LATER framework to identify TSS-TES couplings. Accurate isoform quantification is achieved through the filtering of full-length reads (Figure 6). Comparing observed and expected distributions of full-length 5′–3′ isoforms enables the identification of transcriptome-wide feature couplings (Figures 7A and 7B), as well as the identification of dominant promoters driving transcriptional coupling (Figure 7C). Our method can also be applied to more complex coupling scenarios using LASER, such as alternative splicing events associated with TSS/TES usage (Figure 9). For further information and specific case examples of both LATER and LASER, please refer to our detailed vignette at https://hilgers-lab.github.io/couplings/releases/v1_STAR_protocols/RegulatoryLinks.html.

## Quantification and statistical analysis

### Mapping and read length inspection

**Timing: 2 h**

The following data analysis has been tested extensively on Linux-based servers and computing clusters. We recommend a Linux server with at least 32 GB of memory and 8 CPU cores.

This method uses long-read sequencing libraries to identify transcriptional couplings.

1. Libraries were sequenced on a MinION/PromethION device as described above and FASTQ files were produced using Guppy (guppy-5.0.7 model: dna_r9.4.1_450bps_sup.cfg).
2. FASTQ files were aligned with minimap2 (https://github.com/lh3/minimap2) using the dm6 genome assembly (https://nov2020.archive.ensembl.org/Drosophila_melanogaster). Sorted and indexed using samtools (https://github.com/samtools/samtools).

# Map data to genome using minimap2

 > minimap2 -ax splice -u f genome.fa long_read.fastq.gz | samtools sort -@ 4 -o output.bam

# Index bam file

 > samtools index output.bam

### Long-read estimation of couplings between transcript regions

We developed two computational approaches for processing the alignment files to study transcriptional couplings. LATER (https://github.com/hilgers-lab/LATER) is used for links between 5′ ends and 3′ ends, while LASER (https://github.com/hilgers-lab/LASER) is used for links between 5′ ends with alternative exons and 3′ ends with alternative exons.

Both computational frameworks are based on R.

### Preparation of required files and annotation

3. Install R and all the required packages:

 > install.packages("devtools")

 > devtools::install_github("hilgers-lab/LATER")

 > devtools::install_github("hilgers-lab/LASER")

 > library(LATER)

 > library(LASER)

4. Download the reference annotation for analysis. LATER and LASER build a database that identifies 5′–3′ isoforms that will allow the read to isoform assignment:

 > Annotation_url <- "https://ftp.ensembl.org/pub/release-

109/gtf/drosophila_melanogaster/Drosophila_melanogaster.BDGP6.32.109.gtf.gz"

 > destfile <- "Drosophila_melanogaster.BDGP6.32.109.gtf.gz"

 > download.file(url, destfile)

***Note:*** Utilizing de novo assemblies derived from long-read data has the potential to significantly enhance the outcomes, as it enables the identification of novel 5′–3′ isoforms and increases the detection of isoforms that are not currently annotated.

***Optional:*** A similar procedure can be used for other species available in ensembl (http://www.ensembl.org).

 # Mouse reference annotation

 > Annotation_url <- "https://ftp.ensembl.org/pub/release-

109/gtf/mus_musculus/Mus_musculus.GRCm39.109.gtf.gz"

 > destfile <- "Mus_musculus.GRCm39.109.gtf.gz "

 > download.file(url, destfile)

 # Human reference annotation

 > Annotation_url <- " https://ftp.ensembl.org/pub/release-

109/gtf/homo_sapiens/Homo_sapiens.GRCh38.109.gtf.gz"

 > destfile <- " Homo_sapiens.GRCh38.109.gtf.gz"

 > download.file(url, destfile)

### Estimating transcriptional couplings with 3′ end selection (LATER)

LATER leverages long-read sequencing data to estimate the couplings between transcription start sites (TSSs) and transcription end sites (TESs). It focuses exclusively on full-length reads and identifies genes that exhibit "promoter dominance", whereby a particular TSS is significantly linked to the expression of a specific 3ʹ end.

### Create 5′–3′ isoform database

**Timing: 5 min (for step 5)**

**Timing: 10 min (for step 6)**

5. Use reference annotation to create a 5′–3′ isoform database, this will contain all TSS and TES combinations found in the annotation.

 # load annotation to R

 > refAnnotation <- rtracklayer::import.gff(annot_path)

 # keep only protein coding exons

 > refExons <- refAnnotation[refAnnotation $type == "exon" &

refAnnotation$gene_biotype == "protein_coding"]

 # Prepare isoform database.

 > isoformData <- prepareIsoformDatabase(refExons,

  tss.window=50,

  tes.window=150)

***Note:*** Following parameters have to be tuned depending on the organism or the transcripts of interest. The windows for 5′–3′ isoform definition can substantially affect the couplings detected. *tss.window:* distance in nucleotides to cluster TSSs into a single transcription unit. Depending on the dataset, the parameters for tss.window and tes.window can be optimized. For instance if the resolution of TSS definition is important, tss.window can be reduced to fewer nucleotides and allow quantification of smaller differences. Similarly, for 3′ ends, if windows of less than 150 nt are relevant for the study, the window can be reduced. *tes.window:* distance in nucleotides to cluster TESs into a single TES.

***Optional:*** Many transcriptome annotations, even when up to date, are missing 5′ ends or 3′ ends. This substantially affects the analysis since potential 5′–3′ links will not be considered. To overcome this challenge, it is possible to use already published, highly accurate 3′ end databases as well as TSS/5′ CAGE curated data. Databases like the Eukaryotic Promoter Database (EPD)^2^ can be used to increase the number of annotated TSSs, and hence of 5′–3′ isoforms.

 > url <-

"ftp://ccg.epfl.ch/epdnew/D_melanogaster/005//Dm_EPDnew_005_dm6.bed"

 > file_name <- " dmel_tss_annotation.gtf.gz "

 > download.file(url, file_name)

 > ref_tss_annot <- rtracklayer::import.gff("dmel_tss_annotation.gtf.gz")

***Optional:*** Continuing from the previous optional step, add TSSs databases to the reference annotation.

 > isoformData <- addPromoterDatabase(isoformData,

 ref_tss_annot,

 reference_annotation,

 window = 50)

***Optional:*** A similar approach can be followed to download the 3′ end database. For this protocol, we use the 3′ end database generated in Alfonso-Gonzalez et al., 2023.^1^

 > url <- " https://github.com/hilgers-

lab/CIAtranscriptome_assembly/blob/dk/db/combined.rds.clusters.new.gff"

 > download.file(url, file_name)

 > ref_tes_annot <- rtracklayer::import.gff("dmel_tss_annotation.gtf.gz")

 > isoformData <- add3pEndDatabase(isoformData,

ref_tes_annot,

reference_annotation,

window = 150)

### Quantification of 5′–3′ isoforms

**Timing: 1–2 h**

**Timing: 2–4 h (for optional step in Linux terminal)**

6. After mapping and creating the isoform database, quantify long-reads data.
   > bamPath <- system.file("exdata/testBam.bam", package = 'LATER')
   > countData <- countLinks(bamPath, isoformData)
  ***Note:*** In this step, only reads overlapping a TSS and a TES are counted. The countData object contains all additional information for downstream processing steps.
  ***Optional:*** Write the full-length read id as a tsv file and then use it to subset the mapped bam files using samtools.
  ***Optional:*** In R.
   > readr::write_tsv(readAssignments(countData), "read_assignments.txt")
  ***Optional:*** In Linux terminal:
   > samtools view -N read_assignments.txt -o filtered_output.bam output.bam
7. Long reads can then be visualized using IGV (for an example, see Figure 6).

### Statistical testing of 5′–3′ couplings

**Timing: 30 min (for step 8)**

Our method aims to identify associations between transcription start sites (TSS) and 3′ end sites. By utilizing 5′–3′ isoform counts, our approach determines whether there exists a significant association between the respective expressed TSSs and 3′ ends, within a gene. This analysis involves comparing the observed frequencies of these variables with their expected frequencies, assuming independence between the variables (Figure 7A). For statistical testing, LATER and LASER use multinomial testing. This step requires two objects, the countsData (See “quantification of 5′-3′ isoforms” section) object that contains the counts for every annotated 5′-3′ isoform, and the isoformData (See “create 5′-3′ isoform database” section) object that contains all the information from the annotation.

8. Perform multinomial testing using 5′–3′ isoform counts.

 > gene_bias_estimates <- estimatePromoterDominance(countData, isoformData,

method="chisq")

 # Access resuls from the statistical testing at gene level

 > result(gene_bias_estimates)

 # Access resuls from the statistical testing at isoform level

 > stats(gene_bias_estimates)

 > dominance(gene_bias_estimates)

***Note:*** The assessment of TSS-TES couplings is limited to genes that exhibit multiple transcription start sites (TSS) and transcription end sites (TES). Consequently, the outcomes of this analysis are influenced by both the coverage of the data and the complexity of the transcriptome within the evaluated sample.

9. The overall distribution genome-wide and at individual genes can be visualized with the following functions (for an example, see Figures 7B and 7C).

**Figure 8:**
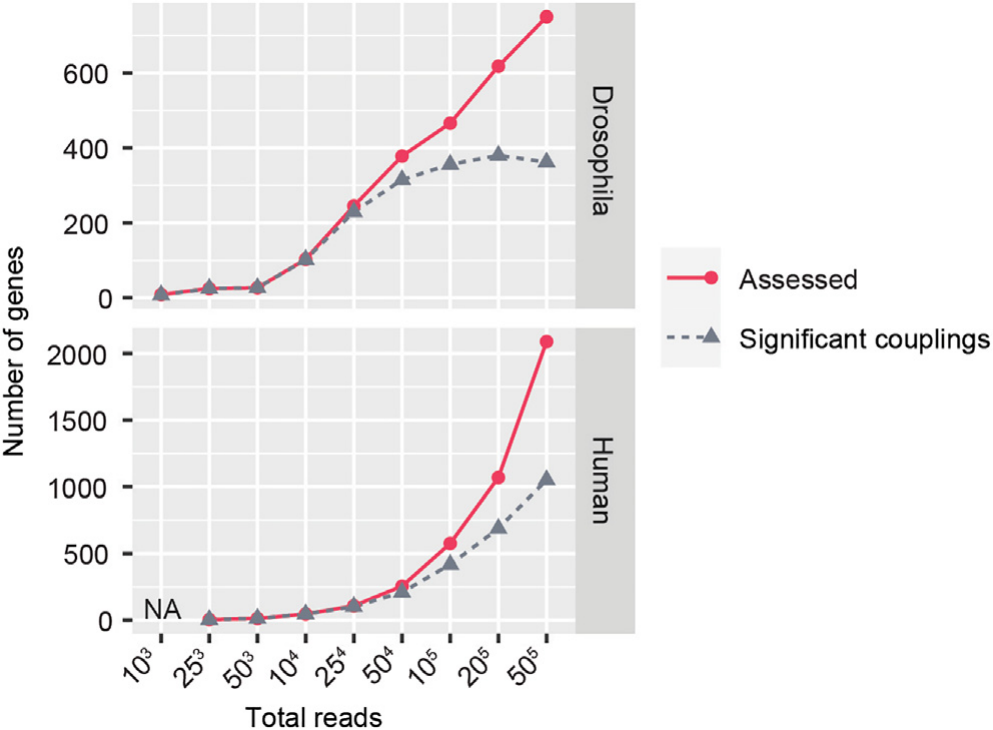
Detection of Regulatory Links as a function of the number of full-length reads Shown are the number of genes that could be assessed (red line), and the number of genes identified as containing significant couplings (gray dotted line).

 # Plot transcriptome-wide

 > plotTranscriptionalBias(gene_bias_estimates,

 xlim =2.5,

 ylim=2.5,

 residual= 0.5,

 showGenes = FALSE)

 # Plot gene with promoter dominance

 > plotGeneBias(gene_bias_estimates, "FBgn0011672")

 # Plot gene with no significant TSS bias

 > plotGeneBias(gene_bias_estimates, "FBgn0004611")

***Note:*** To obtain accurate estimates for transcriptional couplings, high coverage is necessary (Figure 8). Since most reads were discarded after the full-length filtering steps, reads were pooled within conditions.

### Estimating exon couplings with 3′ end/TSS usage (LASER)

In long-read sequencing data, each read contains all the information, starting from the transcription start site (TSS), spanning all exon junctions, and ultimately the 3′ end site. This enables the quantification of co-occurrence frequencies within individual RNA molecules, specifically capturing the relationships between exon junctions, TSS, and TES features (troubleshooting 3).

### Create a junction database

**Timing: 45 min (for step 10)**

**Timing: 2–4 h (for step 11)**

**Timing: 40 min–1.5 h (for step 12)**

**Figure 9:**
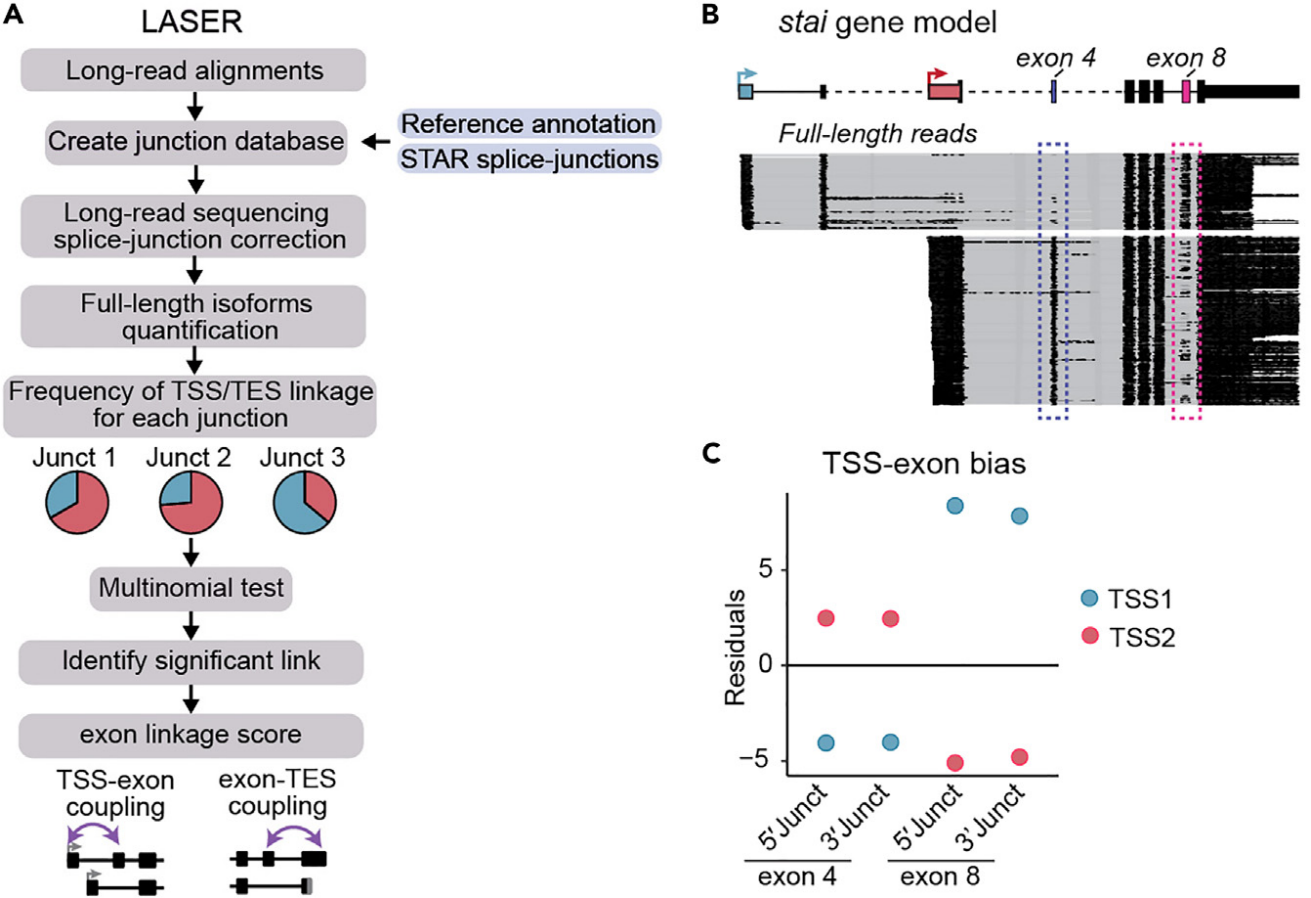
LASER quantifies TSS-exon and exon-TES couplings (A) LASER workflow. (B) Representative example of the *stai* gene demonstrating TSS-exon couplings. Nanopore full-length reads are shown in black, while different colors represent the assessed TSSs. Boxes highlight exons undergoing TSS coupling. (C) Scatter plot illustrating the residuals, which indicate the difference between observed and expected counts. Exon 4 exhibits a significant association with TSS2, whereas exon 8 demonstrates a pronounced bias towards TSS1.

LASER utilizes a reference junction set. This reference is constructed by combining information from a reference annotation and junctions derived from short-read sequencing data obtained through STAR (Figure 9A). The LASER framework provides an example of the format for reference splice junctions, which can be used to obtain the reference junctions from STAR.

10. Create reference junction database using short-read sequencing data and reference annotation.

 > annot_path <- system.file("exdata/dm6.annot.gtf.gz", package="LASER")

 > ref_annot <- rtracklayer::import.gff(annot_path)

 > junction_path <- system.file("exdata/short_read_junctions.SJ.out.tab",

package = 'LASER')

 > reference_junctions <- create_reference_junctions( junction_path,

min.jcounts = 2 , ref_annot, type="short")

***Note:*** We recommend the use of short-read sequencing data to increase the detection of junctions not found in reference annotation.

***Note:*** Depending on the type of data, the following parameters have to be adjusted:

min.jcounts: refers to the minimum number of junction reads from short-read sequencing data for a junction to be considered part of the reference. This parameter is important since lowly expressed or rare variants can substantially affect the analysis.

type: the "short" flag will consider both the reference annotation and short-read sequencing found junctions to build a reference that will be used to correct the long-read sequencing data.

11. Create a full-length read database. Assign reads to a TSS, 3′ end site and junctions found in the database.

 > bamPath <- system.file("exdata/testBam.bam", package = 'LASER')

 > exonlinks.counts<- read_to_junctions(bamPath, reference_junctions,

annot_path)

***Note:*** During this step, reads are filtered to retain only full-length read isoforms that span the transcript from the 5′ to the 3′ end.

***Note:*** Long-read sequencing data are susceptible to a high rate of mismatches, which may result in noisy estimations of exon boundaries. To address this, LASER leverages all the junctions identified in the reference annotation, as well as the short-read data, to correct the long reads and mitigate the impact of mismatches.

12. Perform multinomial testing to identify exon couplings. Additionally, LASER contains gene-level visualization functions (Figure 9C).

 > couplings <- calculate_exon_couplings(exonlinks.counts,

reference_junctions)

 # Plot stai gene couplings

 # Plot couplings exon with TSS

 > plotExonCouplings(couplings, " FBgn0266521", type ="TSS")

 # Plot couplings exon with TES

 > plotExonCouplings(couplings, "FBgn0067779", type = "TES")

***Note:*** Similar to LATER, LASER conducts multinomial testing for every combination of exon junction-TES or exon junction-TES in each full-length molecule. This approach enables the identification of associations between features and to predict meaningful regulatory relationships.

***Note:*** LASER exhibits a high sensitivity to coverage as it relies on an adequate level of splice-junction detection for accurate assessment. Sufficient coverage is crucial to ensure the reliable detection and analysis of splice junctions in order to obtain good results.

## Limitations

The identification of transcriptional couplings is heavily reliant on the coverage and quality of the data. Therefore, it is crucial to have high-quality data to obtain representative and reliable global results. Samples with low RNA quality or yield may not accurately reflect the transcriptome. Furthermore, even with size selection techniques in place, it is not always possible to fully recover transcripts that exceed 10 kb in length.

## Troubleshooting

### Problem 1

Low input total RNA samples.

### Potential solution

• If possible, repeat RNA extractions on biological replicates and pool total RNA fractions.
• For the initial step of the protocol (Isolation of poly(A)+ RNA), 5 μg of total RNA in 50 μL nuclease-free water were used.
• We found that eight poly(A)+ RNA selection reactions (40 μg total RNA) is a good number to start the experiment (including backup).

### Problem 2

Low-quality total RNA samples.

### Potential solution

• If RQN is < 9, the RNA should not be used for full-length analysis.
• A new RNA purification should be performed under RNase-free conditions, with minimal times during which the RNA stays at room temperature.
• Avoid vortexing during the RNA extraction, mix by tube flicking and inverting to avoid RNA damage.

### Problem 3

Computing time too long, more than 7 h.

### Potential solution

• Use run LASER/LATER as batch script. We provide an example using snakemake (https://snakemake.readthedocs.io/en/stable/):

os.getcwd()

localrules: all

# folder containing bam files aligned with minimap2

SAMPLE, = glob_wildcards("workdir/bams_folders/{sample}.bam")

print(SAMPLE)

rule all:

 input:

  expand("LATER_output/{sample}.countedPairs.tsv", sample=SAMPLE)

rule LATER:

  input: "workdir/bams_folders/{sample}.bam",

  ref: "workdir/reference_annotation.gtf"

  output:

   "LATER_output/{sample}.countedPairs.tsv"

  params:

   refAnnot="workdir/reference_annotation.gtf",

   windowTSS=50,

   windowTES=150,

   outfFilePrefix="workdir/countfiles"

  threads: 4

  shell: """

  Rscript LATER.R -b {input[0]} -r {params.refAnnot} -t {params.windowTSS} -

e {params.windowTES} -o {params.outfFilePrefix}"""

• Filter genome for full-length reads using samtools.

>samtools view -b -L tss_regions.bed input.bam | samtools view -b -L pas_regions.bed - > output.bam

## Resource availability

### Lead contact

Further information and requests for resources and reagents should be directed to and will be fulfilled by the lead contact, Valérie Hilgers: hilgers@ie-freiburg.mpg.de.

### Materials availability

This study did not generate new unique reagents.

## Data Availability

• All original code has been deposited at Zenodo and is publicly available. DOIs and GitHub links are listed in the key resources table. • Any additional information required to reanalyze the data reported in this paper is available from the lead contact upon request.
